# Effects of Ursolic Acid on Intestinal Health and Gut Bacteria Antibiotic Resistance in Mice

**DOI:** 10.3389/fphys.2021.650190

**Published:** 2021-05-28

**Authors:** Fang Peng, Haihan Zhang, Xi He, Zehe Song

**Affiliations:** ^1^College of Animal Science and Technology, Hunan Agricultural University, Changsha, China; ^2^Ministry of Education Engineering Research Center of Feed Safety and Efficient Use, Changsha, China

**Keywords:** ursolic acid, anti-inflammation, substitute of antibiotics, ARG, intestine

## Abstract

Ursolic acid (UA), a natural pentacyclic triterpenoid, has been widely reported to exert anti-oxidant and anti-inflammatory properties. However, the effects of UA on the intestinal homeostasis and gut microbiota were rarely explored. The aim of the present study was to investigate the effects of UA on intestinal health and gut microflora antibiotic-resistance in antibiotic-exposed mice. Kunming mice (*n* = 80) were randomly allocated into three groups and fed with one of the following diets, respectively: Cont group (*n* = 20), the basal diet; UA group (*n* = 20), the basal diet supplemented with 150 mg/kg UA; Tet group (*n* = 40), the basal diet supplemented with 659 mg/kg chlortetracycline. After 14 days, 10 mice in each group were euthanatized and the remaining 30 mice in the Tet group were randomly allocated into three sub-groups (*n* = 10 per group) as follows: the Tet group which were kept feeding a Tet diet for 14 days; the Natural Restoration (NatR) group which received a basal diet for 14 days; and the UA therapy (UaT) group which fed a basal diet supplemented with 150 mg/kg UA for 14 days. Throughout the experiment, the weight and the food intake of each mouse were recorded once weekly. Serum LPS and diamine oxidase (DAO), jejunal morphology, jejunal tight junction proteins and nutrient transporters, colonic inflammatory cytokines, gut microbiota and its antibiotic resistance gene (ARG) were examined at euthanasia. The results showed that UA treatment significantly increased average daily food intake (ADFI) of mice. Notably, UA increased the jejunal villi height, decreased the jejunal crypt depth and promoted the expression of jejunum nutrient transporters. UaT group had higher villi height, lower crypt depth and higher nutrient transporter mRNA expression in jejunum than NatR group. Besides, UA decreased serum DAO content, upregulated mRNA expression of ZO-1, claudin-1 and occludin and downregulated TNF-α and IL-6. The mRNA abundances of ZO-1, claudin-1 and occludin and TNF-α and IL-6 in UaT group were, respectively upregulated and downregulated than NatR group. Furthermore, an analysis of 16S rDNA sequences demonstrated that UA increased the abundance of beneficial bacteria in the gut. And the results of ARG test showed that UA downregulated the expression of antibiotic-induced resistance genes. The UaT group inhibited the increase of harmful bacteria abundance and suppressed the mRNA abundances of ARG compared to the NatR group. In conclusion, considering the positive effects of UA on the growth performance and intestinal mucosal barrier, we anticipate that these findings could be a stepping stone for developing UA as a novel substitute of antibiotics.

## Introduction

Subtherapeutic levels of antibiotics have been widely used in the swine and poultry industries to improve weight gain and feed conversion efficiency (Cromwell, 2002; Dibner and Richards, 2005). However, there has been an increasing concern about the health and environmental safety risks of the application of antibiotics, particularly due to the persistence of antibiotic residues and the transmission of antibiotic resistance. For these reasons, the use of antibiotics as feed additives has been restricted or even banned in many countries (Diarra and Malouin, 2014). Recent studies have therefore sought to develop alternative approaches of improving production performance without adverse effects on animal health (Liu et al., 2014; Wang et al., 2020). Among the available alternatives, plant extracts (PEs) or phytogenic additives have been identified as appropriate candidates due to their safety and effective antibacterial behavior (Diaz Carrasco et al., 2016; Ran et al., 2016).

Ursolic acid (UA), a natural pentacyclic triterpenoid, which is widely present in medicinal plants such as bearfruit, hawthorn and fructus ligustri. It has attracted considerable interest in recent years because of various pharmacological activities, such as anti-inflammation and antioxidation. Especially, *in vitro* experiments showed that UA had little or none toxicity to cells (Tohmé et al., 2019). Tan et al. (2017) found that loquat leaf extract which is rich in UA effectively alleviated inflammatory diseases as an natural inhibitor of phospdiesterase-4D (PDE4D). Seow and Lau (2017) reported that UA played a therapeutic role in inflammatory enteritis by regulating the gene expression of progesterone X receptor (PXR). Moreover, UA has been found to inhibit nuclear factor-κB signaling in intestinal epithelial cells and macrophages, and attenuated experimental colitis in mice (Chun et al., 2014; Liu et al., 2016). However, the effects of UA on the gut microbiota structure, intestinal homeostasis and antibiotic resistance is rarely reported. Therefore, the aim of the present study was to investigate the effects of UA on intestinal health and gut flora antibiotic-resistance in antibiotic exposed mice.

## Materials and Methods

### Animal Experiments

The experimental protocol was approved by the Ethics Committee of Hunan Agricultural University (Changsha, China), and every effort was made to minimize animal suffering. Eighty four-week-old male Kunming mice (29–32 g body weight) were purchased from Hunan SJA Laboratory Animal Co., Ltd. (China) and maintained in a designated pathogen-free facility. The mice had access to water and standard chow diet *ad libitum* and were maintained in an environment with a 12:12 h light/dark cycle, a room temperature of 22 ± 2°C, and 55 ± 5% humidity.

**FIGURE 1:**
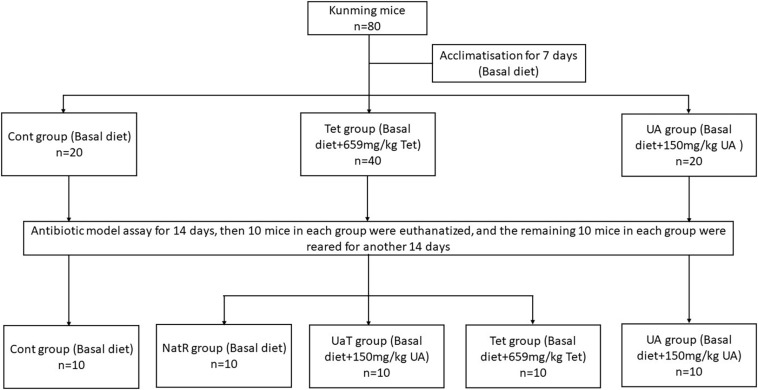
Animal experimental design. Control group (Cont), Chlortetracycline group (Tet), Ursolic acid group (UA), Natural group (NatR), Ursolic acid Therapy group (UaT). The effective concentration of ursolic acid and chlortetracycline was ≥15%.

After a 1-week acclimatization period, the mice were randomly allocated into three groups (Figure 1). Each group was fed one of the following diets, respectively: Cont group (*n* = 20), the basal diet; UA group (*n* = 20), the basal diet supplemented with 150 mg/kg UA; Tet group (*n* = 40), the basal diet supplemented with 659 mg/kg chlortetracycline. After 14 days, 10 mice in each group were killed and the remaining 30 mice in the Tet group were randomly allocated into three sub-groups (*n* = 10 per group) as follows: the Tet group which were kept to feed a Tet diet for 14 days; the Natural Restoration (NatR) group which received a basal diet for 14 days; and the UA Therapy (UaT) group which fed a basal diet supplemented with 150 mg/kg UA for 14 days. The basal diet consists of protein, fat, carbohydrate, fiber, vitamin, and mineral (Supplementary Table 1).

### Sample Collection

Throughout the experiment, the weight and the food intake of each mouse were recorded once weekly. On days 14 and 28 of the experiment, blood samples were collected from the orbital plexus before the mice were sacrificed. Blood samples (1.5 mL) were individually collected from the mouse and then were centrifuged at 4,000 r/min for 15 min at 4°C to obtain serum. At euthanasia, tissues including the jejunum and colon were excised and frozen immediately in liquid nitrogen before further analysis. The colon chyme samples were collected and transferred into sterile precooled tubes, and then stored at -80°C.

### Jejunal Morphology Analysis

Jejunum tissue from mice (*n* = 6) were preserved in 4% paraformaldehyde prior to being dehydrated, embedded in paraffin, sectioned, and stained with hematoxylin and eosin (H&E). Two sections were taken from each intestinal tube, and 10 typical villi heights (VH) and crypt depths (CD) were selected for measurement in each section with Olympus AX70 microscope (Olympus Corporation, Tokyo, Japan). Thereafter, the ratio of VH to CD (VH:CD) was calculated. Intestinal morphological examinations were carried out similar to the procedures described by Jazi et al. (2018). All morphological parameters were measured using the ImageJ Software Package (National Institutes of Health, Bethesda, MD, United States).

### Enzyme-Linked Immunosorbent Assay

The LPS and DAO levels of serum were determined using ELISA kits (Enzyme-linked Biotechnology, Shanghai, China) according to the manufacturer’s instructions. Six mice were tested for each group (*n* = 6).

### Real-Time RT-PCR Analyses

Total RNA was collected and extracted from jejunum and colon samples (*n* = 6) using a RNA rapid extraction kit (CarryHelix Biotechnology, Beijing, China) and transcribed into cDNA using the Primescript RT master mix kit (Takara Bio Inc., Dalian, China). The PCR primer sequences utilized for the determination of the gene expression are shown in Table 1. Gene expression was determined on a real-time quantitative PCR system (CFX Connect; Bio-Rad), using SYBR^®^ Premix Ex Taq^TM^ II (Takara Bio Inc., Shiga, Japan). Relative quantification of the target gene expression was quantified using the 2^–△^^△^^*CT*^ method (Livak and Schmittgen, 2001) and normalized to the expression of Cont group.

**TABLE 1:** Primers used for real-time PCR analysis*.

Gene name	Primer sequence (5′ to 3′)	Product size, bp	Accession number
β-actin	F: AGCTGAGAGGGAAATCGTGC	187	NM_007393.5
	R: GGAAGGCTGGAAAAGAGCCT		
TNF-α	F: ATGGCCTCCCTCTCATCAGT	97	NM_013693.3
	R: TTTGCTACGACGTGGGCTAC		
IL-6	F: AGTCCTTCCTACCCCAATTTCC	79	NM_031168.2
	R: GGTCTTGGTCCTTAGCCACT		
ZO-1	F: GGAGCAGGCTTTGGAGGAG	163	NM_001163574.1
	R: TGGGACAAAAGTCCGGGAAG		
Occludin	F: CCTCCACCCCCATCTGACTA	79	NM_001360536.1
	R: GCTTGCCATTCACTTTGCCA		
Claudin-1	F: CAACCCGAGCCTTGATGGTA	72	NM_016674.4
	R: TCATGCCAATGGTGGACACA		
SGLT1	F: ATGTCTCACGTGAAGGCTGG	156	NM_019810.4
	R: TGGTGTGCCGCAGTATTTCT		
GLUT2	F: GATCACCGGAACCTTGGCTT	76	NM_031197.2
	R: CACACCGATGTCATAGCCGA		
EAAT2	F: AGCCAGTGCACTTCTACAGC	76	NM_001077515.2
	R: CATGCATGCGCACTTCTACC		
EAAT3	F: TTTCTCTCTAGGGGCAGGCT	140	NM_148938.3
	R: CAGAAGGGAGFFCCTCTAGT		
ASCT2	F: TCTGTGGTGTGTGTGCACTT	164	NM_009201.2
	R: AGACTCTAGGGCCATGGTCAA		
B^0^AT1	F: GCTCCTGGACACAACCACTT	133	NM_028878.4
	R: CCAGAGATGGAATCCGCTCC		

*SGLT1, Sodium/glucose cotransporter 1; GLUT2, Glucose transporter 2; EAAT, Excitatory amino acid transporter; ASCT2, Alanine-serine-cysteine transporter 2; B^0^AT1, Broad neutral (0) amino acid transporter 1.

### 16S Ribosomal DNA Gene Sequencing

Since each mouse had very little colonic chyme, two samples were pooled (*n* = 5). The metagenomic DNA was extracted from the colonic chyme samples utilizing the E.Z.N.A.^®^ Stool DNA Kit (Omega Bio-Tek, Norcross, GA, United States) according to the manufacturer’s instructions. The concentrations of the obtained DNA were determined by 1% agarose gel electrophoresis and Nanodrop spectrophotometry (Thermo Fisher Scientific, New York, United States). Then the V3–V4 hypervariable region of the bacterial 16S rDNA gene was PCR amplified with indexed primers (338F: 5′-ACT CCT ACG GGA GGC AGC AG-3′ and 806R: 5′-GAC TAC CVG GGT ATC TAA T-3′) using TransStart^®^ FastPfu DNA Polymerase (TransGen Bitech, Beijing, China). The amplicons were then purified by gel extraction and quantified using a TIANNAMP Soil DNA Kit (TIAGEN, Beijing, China). The purified PCR products were used for high-throughput sequencing using an Illumina MiSeq platform, which was carried out by Personal Biotechnology Co., Ltd., Shanghai, China. The resulting sequences were analyzed using Quantitative Insights into Microbial Ecology. All the sequences were clustered into operational taxonomic units (OTUs) based on a 97% identity threshold by the SILVA database. A representative sequence from each OTU was selected for downstream analysis, and the community richness and diversity indices were calculated.

### Detection of Antibiotic Resistance Genes

For antibiotic resistance genes (ARGs) analysis, the ARGs qRT-PCR array experiments were conducted at Wcgene Biotechnology Corporation, Shanghai. Total RNAs, including ARGs, were isolated from 100 μl of liquid sample (*n* = 6), using a 1-step acidified phenol/chloroform purification protocol. Synthesized exogenous RNAs were spiked into each sample to control for variability in the RNA extraction and purification procedures. The purified RNAs were polyadenylated through a poly(A) polymerase reaction and was then reversed-transcribed into cDNA. Individual ARGs were quantified in real-time SYBR Green RT-qPCR reactions with the specific MystiCq ARGs qPCR Assay Primers (Sigma-Aldrich). The protocol of ARGs qRT-PCR array analysis was as described in detail on the website of Wcgene^1^.

### Statistical Analysis

Data are presented as means ± standard deviation (SD). Shapiro-Wilk was used to test whether the data fit a normal distribution. Thereafter, Levene’s test and one-way analysis of variance (ANOVA) was performed using SPSS 16.0 Software. Mean values of treatment groups were compared using Duncan’s multiple range test with *P* < 0.05 considered statistically significant.

## Results

### Growth Performance

Growth performance of mice at two stages are presented in Table 2. Over day 1–14, mice in the UA group had a higher average daily food intake (ADFI) than Cont group and a higher average daily gain (ADG) and ADFI than Tet group (*P* < 0.05). Over day 15–28, chlortetracycline supplementation significantly reduced (*P* < 0.05) the ADFI and feed-to-gain ratio (F/G) compared with the Cont group, while no significant differences were observed between Cont group and UA group. Furthermore, compared with the NatR group, the UaT group significantly reduced the F/G (*P* < 0.05), and there was no significant difference in F/G between the Tet and UaT groups over the days 15–28 (*P* > 0.05).

**TABLE 2:** Effects of ursolic acid and chlortetracycline on mice average daily gain (ADG), average daily food intake (ADFI), and feed-to-gain ratio (F/G).

Days	Items	Cont	UA	Tet	NatR	UaT
1–14	ADG (g/d)	0.42 ± 0.07^ab^	0.49 ± 0.05^a^	0.37 ± 0.11^b^	–	–
	ADFI (g/d)	7.88 ± 0.88^b^	8.86 ± 0.84^a^	7.20 ± 0.65^b^	–	–
	F/G	18.76 ± 2.10	18.08 ± 1.05	19.46 ± 4.41	–	–
15–28	ADG (g/d)	0.17 ± 0.01	0.21 ± 0.04	0.18 ± 0.05	0.17 ± 0.02	0.17 ± 0.02
	ADFI (g/d)	8.53 ± 0.53^b^	9.27 ± 0.36^ab^	7.02 ± 0.53^c^	9.82 ± 1.22^a^	6.85 ± 0.55^c^
	F/G	50.18 ± 1.26^b^	44.14 ± 7.21^bc^	39.00 ± 8.61^c^	57.76 ± 7.00^a^	40.29 ± 1.47^c^

*^*a,b*^Different letters in the same row indicate significant differences between the respective means (P < 0.05).*

*Days 1–14, Cont, basal diet for 2 weeks (control); UA, basal diet supplemented with 150 mg/kg ursolic acid for 2 weeks; Tet, basal diet supplemented with 659 mg/kg chlortetracycline for 2 weeks; Days 15–28, Cont, basal diet for 4 weeks (control); UA, basal diet supplemented with 150 mg/kg ursolic acid for 4 weeks; Tet, basal diet supplemented with 659 mg/kg chlortetracycline for 4 weeks; NatR, basal diet supplemented with 659 mg/kg chlortetracycline for 2 weeks and then basal diet for 2 weeks; UaT, basal diet supplemented with 659 mg/kg chlortetracycline for 2 weeks and then basal diet supplemented with 150 mg/kg ursolic acid for 2 weeks.*

### Intestinal Morphology

**FIGURE 2:**
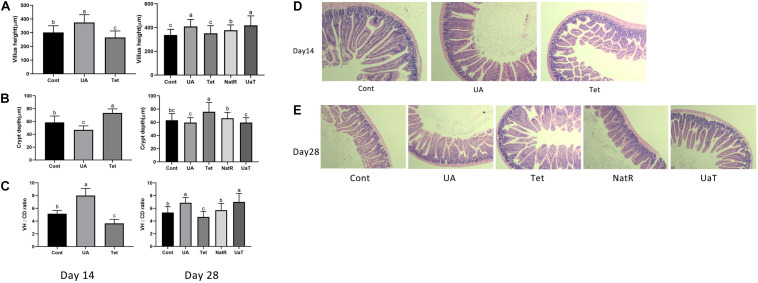
Administration of ursolic acid attenuates antibiotic-induced jejunal morphological damage. Villus height **(A)**, crypt depth **(B)**, and VH: CD **(C)** in the jejunum of mice. Histology picture of jejunum in mice **(D,E)**. Day 14: Control (Cont) indicates the Non-treatment group, Chlortetracycline (Tet) indicates basal diet supplemented with 659 mg/kg chlortetracycline for 2 weeks, Ursolic acid (UA) indicates basal diet supplemented with 150 mg/kg ursolic acid for 2 weeks. Day 28: Control (Cont) indicates the Non-treatment group, Chlortetracycline (Tet) indicates basa diet supplemented with 659 mg/kg chlortetracycline for 4 weeks, Ursolic acid (UA) indicates basal diet supplemented with 150 mg/kg ursolic acid for 4 weeks, Natural restoration (NatR) indicates basal diet supplemented with 659 mg/kg chlortetracycline for 2 weeks and then feed with basal diet, Ursolic acid therapy (UaT) indicates basal diet supplemented with 659 mg/kg chlortetracycline for 2 weeks and then supplemented with 150 mg/kg ursolic acid for 2 weeks. Data are shown as means ± standard deviations. Statistically significance results between each treatment group on days 14 and 28 were expressed by lowercase letters (a,b,c) based on ANOVA with Duncan’s range tests. Mean values with different letters indicate statistically significance results (*P* < 0.05). *n* = 6 mice per group.

The intestinal morphology results are shown in Figure 2. On days 14 and 28, the UA group had markedly higher jejunal VH and VH:CD ratio compared to the Cont and Tet groups (*P* < 0.05), and the Tet group had significantly higher CD but lower jejunal VH and VH:CD ratio than Cont group (*P* < 0.05). Jejunal CD in the UA group was shorter (*P* < 0.05) than Cont group on day 14, but there was no significant difference between the two groups on day 28. On day 28, compared to the Tet group, mice in the NatR and UaT groups had higher jejunal VH and VH:CD ratio and lower jejunal CD (*P* < 0.05). Moreover, the UaT group had significantly lower CD and greater VH and VH:CD ratio in the jejunum compared to the NatR group on day 28 (*P* < 0.05).

### Serum LPS and DAO Levels

**FIGURE 3:**
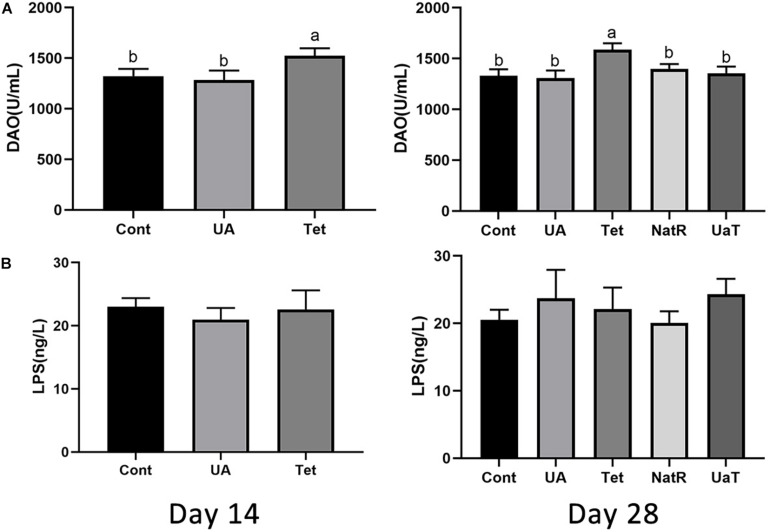
Administration of ursolic acid mitigates antibiotic-induced gut barrier dysfunction. Levels of DAO **(A)** and LPS **(B)** in the serum of mice. All group names correspond to those described in the legend of Figure 2. Data are shown as means ± standard deviations. Statistically significance results between each treatment group on days 14 and 28 were expressed by lowercase letters (a,b,c) based on ANOVA with Duncan’s range tests. Mean values with different letters indicate statistically significance results (*P* < 0.05). *n* = 6 mice per group.

Gut permeability was indicated by the levels of LPS and DAO in the serum, which are shown in Figure 3. On days 14 and 28, DAO levels were significantly reduced in the UA and Cont group vs. the Tet group (*P* < 0.05), and no significant difference was observed between the UA and Cont groups. Furthermore, the Tet group had markedly higher serum DAO levels vs. the NatR and UaT groups (*P* < 0.05), and there were no significant differences of DAO levels between the NatR and UaT groups on day 28 (*P* > 0.05). However, no significant difference was observed in the concentrations of serum LPS among all the groups either on days 14 or 28 (*P* > 0.05).

### Gene Expression of Jejunal Epithelial Tight-Junction Proteins

**FIGURE 4:**
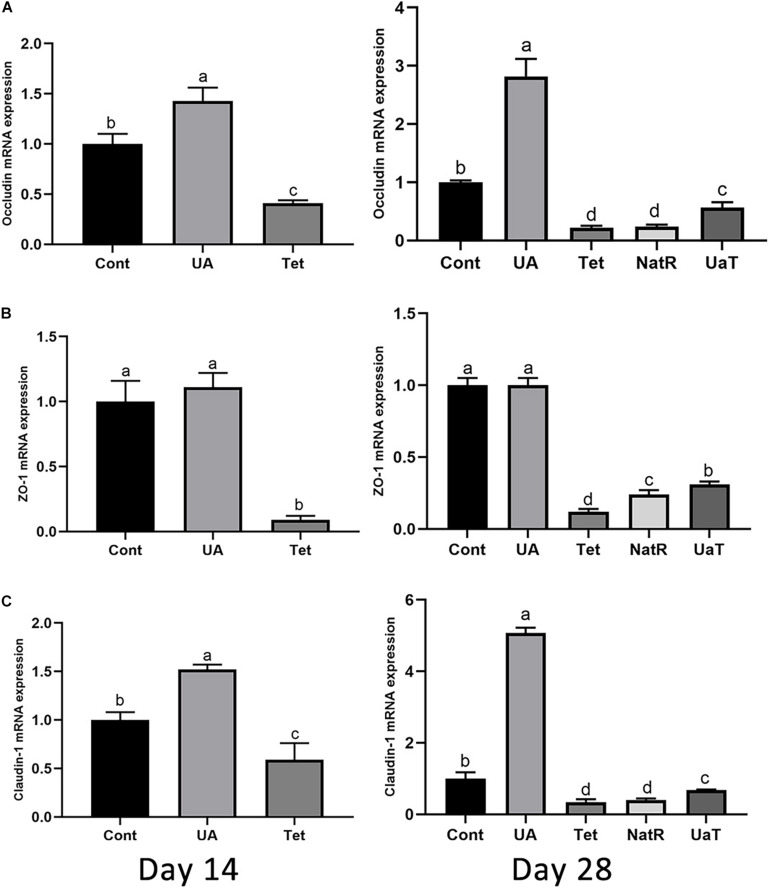
Effects of administration of ursolic acid on the expression of mRNAs corresponding to the tight-junction proteins occludin **(A)**, ZO-1 **(B)**, and claudin-1 **(C)** in jejunum tissue of mice. All group names correspond to those described in the legend of Figure 2. Data are shown as means ± standard deviations. Statistically significance results between each treatment group on days 14 and 28 were expressed by lowercase letters (a,b,c) based on ANOVA with Duncan’s range tests. Mean values with different letters indicate statistically significance results (*P* < 0.05). *n* = 6 mice per group.

The gene expression of jejunal epithelial tight-junction proteins, including ZO-1, occludin and claudin-1, which are shown by Figure 4. On days 14 and 28, occludin and claudin-1 mRNA expression were upregulated in UA group vs. the Cont and Tet groups (*P* < 0.05), and the Tet group significantly downregulated the tight-junction proteins (ZO-1, occludin, claudin-1) mRNA expression relative to the Cont group (*P* < 0.05). Besides, the ZO-1 mRNA expression in UA group had no significant difference from that in Cont group on days 14 and 28 (*P* > 0.05). On day 28, in comparison to the Tet and NatR groups, the UaT group had higher mRNA expression levels of the tight-junction proteins (ZO-1, occludin, claudin-1) (*P* < 0.05), and no significant difference between the Tet and NatR groups were observed in mRNA abundance of occludin and claudin-1 (*P* > 0.05) except ZO-1 which were upregulated in the NatR group (*P* < 0.05).

### Gene Expression of Jejunal Epithelial Amino Acid and Glucose Transporters

As presented in Table 3, compared to the Cont group, UA supplementation led to an augment in jejunal B^0^AT1, EAAT2, EAAT3, and SGLT1 mRNA expression while treatment with chlortetracycline significantly increased EAAT2, EAAT3, and SGLT1 mRNA expression but decreased the mRNA expression of B^0^AT1 (*P* < 0.05) on day 14. Compared with the Tet group, UA supplementation significantly upregulated the mRNA expression of B^0^AT1, EAAT2 and SGLT1 but downregulated the mRNA expression of EAAT3 on day 14 (*P* < 0.05). On day 14, the UA group significantly reduced jejunal GLUT2 mRNA expression relative to the Cont group (*P* < 0.05), and there was no obvious difference between the UA and Tet groups. On day 28, all amino acid transporters (B^0^AT1, ASCT2, EAAT2, EAAT3) mRNA expression in the NatR and UaT groups were downregulated relative to the Tet group, but compared to the NatR group, the UaT group had greater mRNA abundances of B^0^AT1, ASCT2, EAAT3, and SGLT1 (*P* < 0.05). On day 28, no remarkable difference in jejunal GLUT2 mRNA expression was observed among the Cont, UA and Tet groups. Furthermore, the NatR group had higher GLUT2 mRNA expression in the jejunum than Tet and UaT groups (*P* < 0.05) and no statistically supported difference was observed between the Tet and UaT groups.

**TABLE 3:** Gene expression of nutrient transporter in the jejunum of mice^1^.

	Item	Cont	UA	Tet	NatR	UaT
Day 14	B^0^AT1	1 ± 0.2^b^	1.26 ± 0.13^a^	0.26 ± 0.02^c^	–	–
	ASCT2	1 ± 0.21	1.08 ± 0.03	0.96 ± 0.10	–	–
	EAAT2	1 ± 0.1^c^	2.67 ± 0.4^a^	1.87 ± 0.13^b^	–	–
	EAAT3	1 ± 0.18^c^	3.99 ± 0.43^b^	6.02 ± 0.45^a^	–	–
	GLUT2	1 ± 0.22^a^	0.78 ± 0.15^b^	0.82 ± 0.05^ab^	–	–
	SGLT1	1 ± 0.08^c^	1.87 ± 0.26^a^	1.57 ± 0.23^b^	–	–
Day 28	B^0^AT1	1 ± 0.2^bc^	0.53 ± 0.03^d^	3.83 ± 0.5^a^	0.85 ± 0.12^cd^	1.25 ± 0.3^b^
	ASCT2	1 ± 0.17^d^	2.24 ± 0.06^a^	2 ± 0.14^b^	1.06 ± 0.23^d^	1.73 ± 0.25^c^
	EAAT2	1 ± 0.09^c^	4.78 ± 0.54^a^	2.94 ± 0.78^b^	1.03 ± 0.15^c^	1.27 ± 0.12^c^
	EAAT3	1 ± 0.19^d^	1.56 ± 0.27^d^	9.73 ± 0.53^a^	7.59 ± 0.56^c^	8.34 ± 1.08^b^
	GLUT2	1 ± 1.29^b^	0.64 ± 0.05^b^	0.75 ± 0.14^b^	11.69 ± 0.4^b^	1.04 ± 0.16^b^
	SGLT1	1 ± 1.64^c^	4.39 ± 0.47^b^	6.28 ± 1.04^a^	1.2 ± 0.11^c^	7.12 ± 1.21^a^

*^1^Each value represents the mean of six replicates.*

*^*a,b*^Different letters in the same row indicate significant differences between the respective means (P < 0.05).*

*Days 1–14, Cont, basal diet for 2 weeks (control); UA, basal diet supplemented with 150 mg/kg ursolic acid for 2 weeks; Tet, basal diet supplemented with 659 mg/kg chlortetracycline for 2 weeks; Days 15–28, Cont, basal diet for 4 weeks (control); UA, basal diet supplemented with 150 mg/kg ursolic acid for 4 weeks; Tet, basal diet supplemented with 659 mg/kg chlortetracycline for 4 weeks; NatR, basal diet supplemented with 659 mg/kg chlortetracycline for 2 weeks and then basal diet for 2 weeks; UaT, basal diet supplemented with 659 mg/kg chlortetracycline for 2 weeks and then basal diet supplemented with 150 mg/kg ursolic acid for 2 weeks.*

### Gene Expression of Colonic Inflammatory Cytokines

**FIGURE 5:**
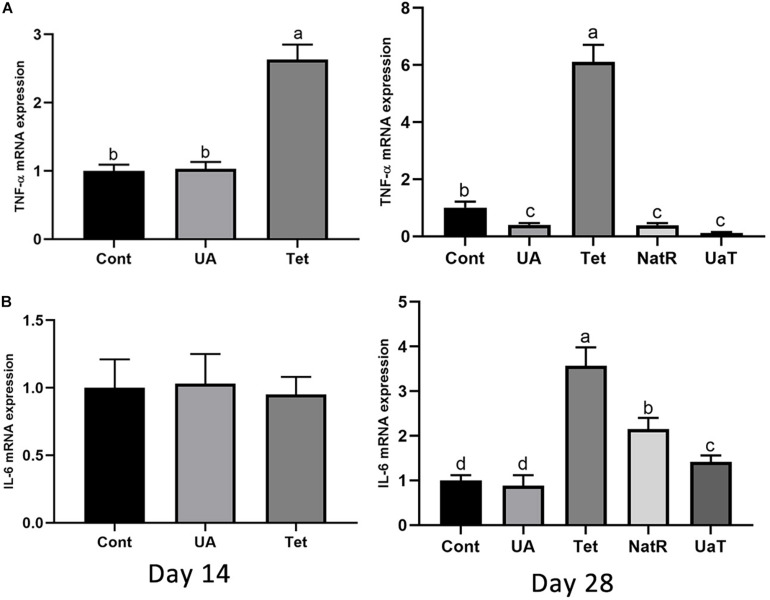
Effects of administration of ursolic acid on the expression of mRNAs corresponding to TNF-α **(A)** and IL-6 **(B)** in colon tissue of mice. All group names correspond to those described in the legend of Figure 2. Data are shown as means ± standard deviations. Statistically significance results between each treatment group on days 14 and 28 were expressed by lowercase letters (a,b,c) based on ANOVA with Duncan’s range tests. Mean values with different letters indicate statistically significance results (*P* < 0.05). *n* = 6 mice per group.

As shown in Figure 5, the Tet group increased colonic TNF-α mRNA expression on day 14 and 28 vs. the Cont and UA groups (*P* < 0.05). There was no significant difference in TNF-α mRNA expression of colon between the Cont and UA groups on day 14 but UA treatment downregulated TNF-α expression in colon on day 28 (*P* < 0.05). The NatR and UaT groups remarkably downregulated (*P* < 0.05) increased colonic TNF-α mRNA expression induced by chlortetracycline treatment, but there was no significant difference between NatR and UaT groups (*P* > 0.05). In addition, no significant difference was observed in colonic IL-6 mRNA expression among groups on day 14, but the Tet group had markedly increased the gene expression of IL-6 on day 28 relative to Cont and UA groups (*P* < 0.05). Furthermore, the mRNA abundance of IL-6 in UA group had no statistically supported difference from that in Cont group on day 28 (*P* > 0.05). In comparison to the Tet group, the NatR and UaT groups markedly downregulated the mRNA expression of IL-6 (*P* < 0.05), and the UaT group had lower mRNA abundance of IL-6 relative to the NatR group (*P* < 0.05).

### Diversity and Structure of Intestinal Microbiota

**FIGURE 6:**
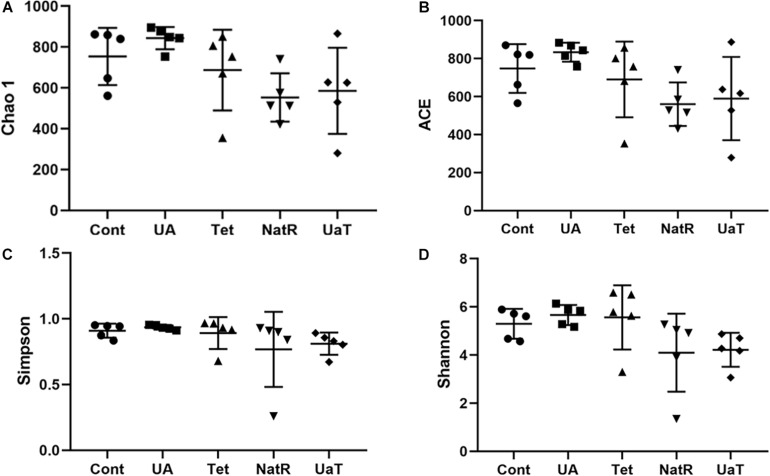
Analysis of alpha-diversity in the five experimental groups on day 28. Chao1 **(A)** and ACE **(B)** were used as richness estimators. Simpson **(C)** and Shannon **(D)** were used as diversities estimators. All group names correspond to those described in the legend of Figure 2.

**FIGURE 7:**
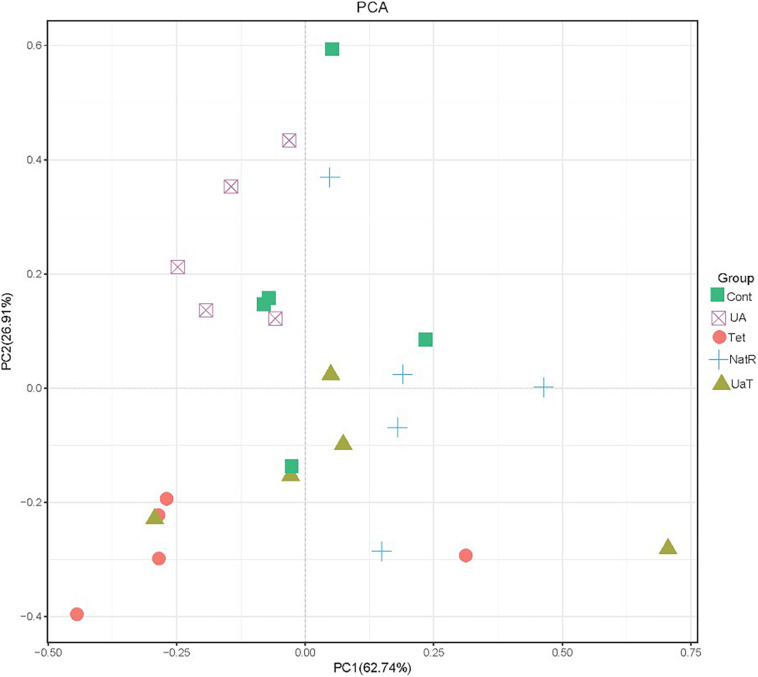
Principal component analysis (PCA) of the structure of the gut microbiota. All group names correspond to those described in the legend of Figure 2.

The Chao 1 and ACE indices that estimate microbial richness, and the Shannon and Simpson diversity indices which reflects species biodiversity, were calculated to evaluate the alpha diversity (Figure 6). On day 28, there were no significant differences in the colonic microbiota alpha diversity among the three groups (Cont, UA, Tet) and the other three groups (Tet, NatR, UaT). Principal component analysis (PCA) indicated that microbes from the colon of the UA and Tet groups formed distinct clusters. No differences in colonic microbial communities among the Tet, NatR, and UaT groups were identified on day 28 (Figure 7).

### Relative Abundance of Bacterial Taxa in the Intestinal Microbiota

**FIGURE 8:**
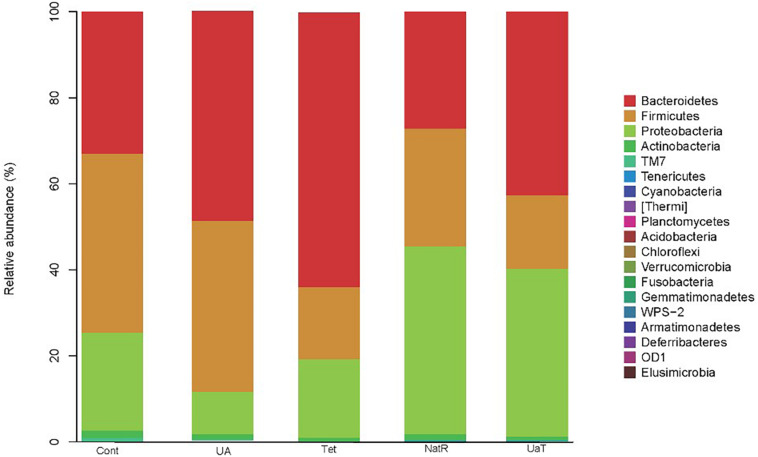
The relative abundances of the colonic microbiota at the phylum levels. The relative abundances of the gut bacteria presented here were calculated by averaging the data obtained from the five replicates within each group. All group names correspond to those described in the legend of Figure 2.

At the phylum level, Bacteroidetes, Firmicutes, Proteobacteria, and Actinobacteria were the dominating colonic microbial community in all five groups (Figure 8). Compared with the Cont and Tet groups, the relative abundance of Proteobacteria in UA group was reduced (*P* < 0.05). The treatment with chlortetracycline reduced relative abundance of Firmicutes but increased relative abundance of Bacteroidetes. Additionally, the relative abundances of Proteobacteria in NatR and UaT groups were increased compared to the Tet group, and the relative abundance of Proteobacteria in UaT group was less than NatR group (*P* < 0.05).

**FIGURE 9:**
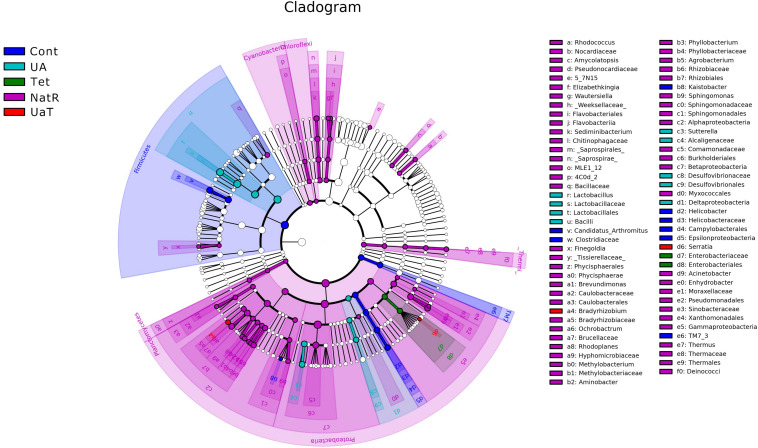
Cladogram of the LEfSe analysis of the gut microbiota in different groups. The microbial compositions were compared at different evolutionary levels. All group names correspond to those described in the legend of Figure 2.

**FIGURE 10:**
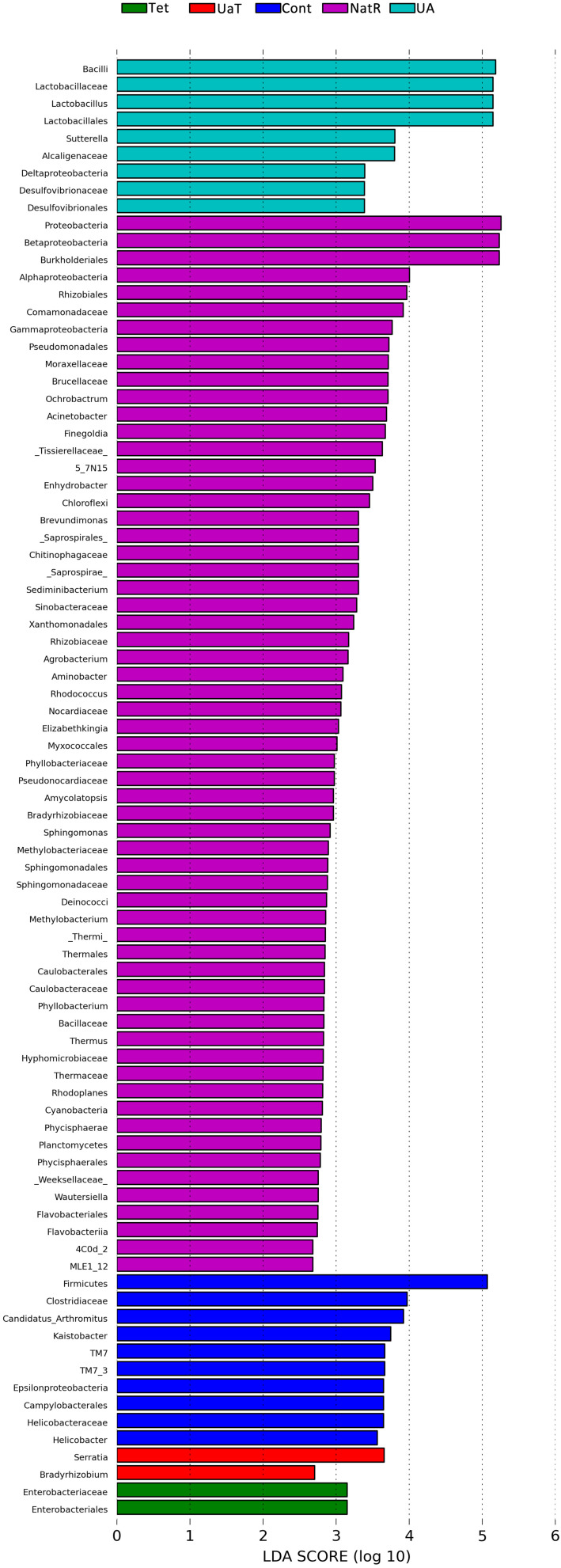
LDA scores obtained from the LEfSe analysis of the gut microbiota in different groups. An LDA effect size of greater than 2 was used as a threshold for the LEfSe analysis. All group names correspond to those described in the legend of Figure 2.

The LEfSe analysis showed a significant enhancement in the abundance of the beneficial bacteria *Lactobacillus* in the UA group. Inversely, the populations of *Enterobacteriaceae* showed a remarkable increase in the Tet group. Besides, more potentially harmful bacteria *Burkholderiales*, *Alphaproteobacteria*, *Betaproteobacteria*, *Gammaproteobacteria* were found in group NatR but not in group UaT (Figures 9, 10).

### Antibiotic Resistance Gene Expression

As shown in Table 4, 44 tetracycline resistance genes (TRGs) in gut digesta were examined, but 14 genes were undetermined in all groups. On day 28, chlortetracycline treatment caused a detectable increase in abundance of TRGs vs. the other groups, and there were three genes [TET(35), TETD-01, TETG-02] only detected in group Tet or in group NatR. Compared to the Tet and Cont groups, the UA group significantly downregulated the TRGs expression of TET(34), TETG-01, and TETL-01 (*P* < 0.05). The NatR and UaT groups had markedly reduced some increased TRGs expression in group Tet, and the UaT group had lower mRNA abundances of TETM-02, TETQ, TETG-01, TETR-03, TETB-02, TET(36)-01, and TETG-02 when compared to the NatR group (*P* < 0.05).

**TABLE 4:** Gene expression of tetracycline resistance genes in gut digesta of mice^1^.

	Item	Cont	UA	Tet	NatR	UaT
Day 28	16S rDNA	16.67 ± 1.09	16.26 ± 0.97	17.48 ± 0.99	16.07 ± 1.57	17.05 ± 1.25
	TET(32)	1.00 ± 0.29^c^	1.43 ± 0.42^c^	3.21 ± 0.46^a^	0.41 ± 0.08^d^	2.13 ± 0.54^b^
	TET(34)	1.00 ± 0.2^bc^	0.20 ± 0.06^d^	0.74 ± 0.16^c^	1.29 ± 0.42^b^	2.67 ± 0.64^a^
	TETA-02	1.00 ± 0.17^b^	0.17 ± 0.03^b^	1373.18 ± 334.46^a^	3.86 ± 0.82^b^	8.39 ± 1.52^b^
	TETB-01	1.00 ± 0.19^bc^	3.01 ± 0.79^a^	0.24 ± 0.03^c^	2.83 ± 1.09^a^	1.64 ± 0.44^b^
	TETC-01	1.00 ± 0.34^bc^	1.50 ± 0.44^b^	0.90 ± 0.18^c^	2.26 ± 0.66^a^	0.03 ± 0.01^d^
	TETC-02	1.00 ± 0.20^b^	0.24 ± 0.06^c^	0.14 ± 0.04^c^	3.27 ± 1.07^a^	0.11 ± 0.03^c^
	TETK	1.00 ± 0.33^ab^	3.13 ± 4.07^a^	0.46 ± 0.12^b^	0.59 ± 0.20^b^	0.12 ± 0.04^b^
	TETL-02	1.00 ± 0.24^bc^	0.09 ± 0.02^d^	0.74 ± 0.26^cd^	3.33 ± 1.00^a^	1.46 ± 0.40^b^
	TETM-01	1.00 ± 0.17^d^	20.94 ± 4.45^cd^	150.16 ± 38.77^a^	56.95 ± 15.37^b^	32.17 ± 8.03^bc^
	TETM-02	1.00 ± 0.32^c^	1.17 ± 0.36^c^	15.64 ± 4.07^a^	13.96 ± 4.57^a^	5.45 ± 1.42^b^
	TETO-01	1.00 ± 0.22^b^	0.82 ± 0.10^b^	5.58 ± 1.57^a^	0.68 ± 0.10^b^	1.22 ± 0.24^b^
	TETO-02	1.00 ± 0.23^b^	0.74 ± 0.12^b^	5.23 ± 1.79^a^	0.24 ± 0.04^b^	0.86 ± 0.21^b^
	TETQ	1.00 ± 0.38^d^	1.75 ± 0.30^c^	5.02 ± 0.65^a^	2.36 ± 0.39^b^	1.36 ± 0.30^cd^
	TETR-01	1.00 ± 0.37^c^	1.7 ± 0.34^bc^	1.73 ± 0.73^bc^	3.14 ± 1.05^a^	2.38 ± 0.90^ab^
	TETR-02	1.00 ± 0.20^b^	0.42 ± 0.05^b^	0.56 ± 0.11^b^	3.85 ± 0.82^a^	4.38 ± 1.30^a^
	TETS	1.00 ± 0.38^b^	0.49 ± 0.13^b^	4.01 ± 0.55^a^	0.89 ± 0.26^b^	4.57 ± 1.10^a^
	TETW-01	1.00 ± 0.40^e^	2.50 ± 0.37^d^	7.20 ± 1.52^b^	4.71 ± 0.70^c^	9.83 ± 1.74^a^
	TETX	1.00 ± 0.33^c^	0.82 ± 0.11^c^	1.93 ± 0.47^b^	2.18 ± 0.57^ab^	2.59 ± 0.55^a^
	TETG-01	1.00 ± 0.34^a^	0.29 ± 0.08^b^	0.73 ± 0.25^a^	0.68 ± 0.15^a^	Undetermined
	TETT	1.00 ± 0.19^b^	0.97 ± 0.30^b^	0.81 ± 0.17^b^	3.69 ± 0.36^a^	0.66 ± 0.19^b^
	TETD-02	Undetermined	Undetermined	Undetermined	Undetermined	Undetermined
	TETR-03	1.00 ± 0.17^c^	1.79 ± 0.41^a^	1.36 ± 0.24^b^	1.46 ± 0.30^ab^	0.59 ± 0.15^d^
	TETL-01	1.00 ± 0.15^c^	Undetermined	2.36 ± 0.59^a^	0.45 ± 0.11^c^	1.58 ± 0.56^b^
	TETB-02	1.00 ± 0.45^c^	0.26 ± 0.06^c^	2.45 ± 0.74^b^	8.37 ± 1.77^a^	Undetermined
	TET(36)-01	1.00 ± 0.23^c^	0.89 ± 0.21^c^	2.58 ± 0.75^a^	1.83 ± 0.30^b^	Undetermined
	TET(35)	Undetermined	Undetermined	17.06 ± 0.48	Undetermined	Undetermined
	TETD-01	Undetermined	Undetermined	19.45 ± 0.26	Undetermined	Undetermined
	TETG-02	Undetermined	Undetermined	13.11 ± 0.31	14.65 ± 0.40	Undetermined
	TETPA	Undetermined	20.04 ± 0.44	Undetermined	Undetermined	Undetermined
	TETB(38)	17.37 ± 0.43	Undetermined	Undetermined	Undetermined	Undetermined
	TETPB-02	18.21 ± 0.39	Undetermined	Undetermined	Undetermined	Undetermined
	TET(36)-02	Undetermined	Undetermined	Undetermined	Undetermined	Undetermined
	TET(37)	Undetermined	Undetermined	Undetermined	Undetermined	Undetermined
	TETA-01	Undetermined	Undetermined	Undetermined	Undetermined	Undetermined
	TETE	Undetermined	Undetermined	Undetermined	Undetermined	Undetermined
	TETH	Undetermined	Undetermined	Undetermined	Undetermined	Undetermined
	TETJ	Undetermined	Undetermined	Undetermined	Undetermined	Undetermined
	TETPB-01	Undetermined	Undetermined	Undetermined	Undetermined	Undetermined
	TETPB-03	Undetermined	Undetermined	Undetermined	Undetermined	Undetermined
	TETPB-04	Undetermined	Undetermined	Undetermined	Undetermined	Undetermined
	TETPB-05	Undetermined	Undetermined	Undetermined	Undetermined	Undetermined
	TETU-01	Undetermined	Undetermined	Undetermined	Undetermined	Undetermined
	TETU-02	Undetermined	Undetermined	Undetermined	Undetermined	Undetermined
	TETV	Undetermined	Undetermined	Undetermined	Undetermined	Undetermined

*^1^Each value represents the mean of six replicates.*

*^*a,b*^Different letters in the same row indicate significant differences between the respective means (P < 0.05).*

*Days 15–28, Cont, basal diet for 4 weeks (control); UA, basal diet supplemented with 150 mg/kg ursolic acid for 4 weeks; Tet, basal diet supplemented with 659 mg/kg chlortetracycline for 4 weeks; NatR, basal diet supplemented with 659 mg/kg chlortetracycline for 2 weeks and then basal diet for 2 weeks; UaT, basal diet supplemented with 659 mg/kg chlortetracycline for 2 weeks and then basal diet supplemented with 150 mg/kg ursolic acid for 2 weeks.*

## Discussion

Sub-therapeutic antibiotics supplementation in the feed can improve the growth performance of animals and increase the economic benefits of breeding industry. However, there are some non-negligible side effects resulting from the use of antibiotics. Epidemiological studies show a relationship between the overuse of antibiotic and an increased risk of dysbiosis and inflammatory diseases (Cho et al., 2012; Canova et al., 2014; Goulet, 2015). In our study, increased risk of intestinal inflammation, bacterial imbalance, and upregulated expression of antibiotic-resistant genes were also observed after antibiotic administration. Interestingly, we observed that the use of UA reversed this dysbiosis. In addition, we found that UA also had a positive effect on growth performance. At present, quite few literatures analyzed and discussed the effect of UA on growth performance. Previous studies have shown that tea saponins, as one of the triterpenoids, had potential in improving the animal growth performance, and the mechanism might be through the regulation of intestinal flora (Hu et al., 2006; Wang et al., 2012). So, we speculate that UA, which also belongs to triterpenoids, may have the same regulatory effect on growth performance of animals. Although it remains unknown how PEs improves animal growth, several mechanisms have been proposed, including modifications of intestinal microbial ecology (e.g., reducing pathogenic stress or increasing the abundance of beneficial microorganism in the gut), increasing digestive enzyme secretions, or improving nutrient absorption (Windisch et al., 2008; Awaad et al., 2014; Wlodarska et al., 2015; Zeng et al., 2015; Stevanović et al., 2018).

The length of villi and the depth of crypt are important morphological parameters, and are considered as indicators of intestinal functions. Although previous studies showed that dietary supplementation with PEs improved the intestinal morphology in mice (Lee et al., 2018; Xue et al., 2020), the effect of UA on intestinal morphology has not been reported yet. In our present study, dietary supplementation with UA increased the jejunal villus height and villus height to crypt depth ratio, decreased the crypt depth in mice. Specially, our results indicated that UA could repair the damage of intestinal morphology caused by antibiotics. Previous studies have shown that the absorption of glucose and amino acids was crucial to the growth and development of animals (Wu, 1998; Chen et al., 2018). In this study, we detected that UA promoted the mRNA expression of glucose transporters SGLT1 but suppressed GLUT2. On the contrary, previous study has shown that UA inhibited the mRNA expression of SGLT1 and GLUT2 in intestinal epithelial cells (Wu et al., 2014). Yin et al. (2019) demonstrated that up-regulated expression of SGLT1 could maintain growth performance and intestinal function of broilers (Yin et al., 2019). Besides, we found that UA promoted the expression of amino acid transporters (B^0^AT1, ASCT2, EAAT2, EAAT3). It is certain that there is a positive correlation between the increased expression of amino acid transporter and the growth performance (Zhang Y. L. et al., 2019; Park et al., 2020). However, there were few reports on the effect of UA on amino acid transporters so far and the underlying mechanisms should be further explored. In general, UA may promote growth performance due to its regulation of intestinal morphology and nutrient transport carriers.

Additionally, the effect of UA on growth performance may also be related to the improvement of intestinal barrier function. In this study, we have demonstrated that the antibiotic administration to mice increased the levels of serum DAO, but dietary supplementation of UA did not. The integrity of tight junction could strengthen the intestinal barrier, thus limiting the ability of antigens to enter the body and preventing adverse immune responses. Supplementation of UA upregulated the expression of three tight junction proteins (occludin, ZO-1, and claudin-1) and downregulated two inflammatory cytokines (TNF-α and IL-6). Consistent with our results, Zhang W. et al. (2019) found that oral administration of 40 mg/kg UA significantly increased the expression of claudin-1 and occludin in the intestinal tissues of rats (Zhang W. et al., 2019). Wan et al. (2019) found that UA prevented the disruption of tight junctions, alleviated inflammation and decreased intestinal permeability in mice with liver fibrosis (Wan et al., 2019). Antibiotic-exposed mice were reported to produce higher levels of pro-inflammatory cytokines, including TNF-α and IL-6 in tissues, than the untreated mice (Suzaki et al., 1999; Knoop et al., 2016). In this study, we also detected increases of TNF-α and IL-6 mRNA expression levels following chlortetracycline treatment. In a recent study, UA downregulated the expression of the TNF-α and IL-6 in immune cells co-cultured with Toxoplasma (Choi and Lee, 2019). Most studies have found that UA had a strong anti-inflammatory activity (Kashyap et al., 2016; Habtemariam, 2019; Rai et al., 2019). Our results are consistent with previous studies, suggesting that UA can maintain homeostasis of intestinal function by enhancing intestinal barrier function and inhibiting intestinal inflammation.

A single dose of antibiotic is sufficient to induce gut microbiota dysbiosis (Knoop et al., 2016). In this study, the use of chlortetracycline caused structural changes in the bacterial flora, although it had no significant effect on colonic microbiota alpha diversity. At phylum levels, chlortetracycline treatment reduced the abundance of Firmicutes but increased the abundance of Bacteroides. The use of chlortetracycline and UA reduced the abundance of Proteobacteria, and UA therapy inhibited the increase of Proteobacteria compare to natural restoration when chlortetracycline was discontinued. We found that commensal bacteria, such as *Lactobacillus*, declined in abundance in the Tet group whereas *Enterobacteriaceae* was enriched in abundance in response to antibiotic exposure. In contrast, the abundance of *Lactobacillus* was increased greatly in the UA group. Besides, LEfSe analysis showed more potentially harmful bacteria such as *Burkholderiales*, *Alphaproteobacteria*, *Betaproteobacteria*, and *Gammaproteobacteria* were increased significantly in group NatR but not in group UaT. All of the above indicated that UA might adjust the structure of bacterial flora by increasing the abundance of beneficial bacteria and decreasing the abundance of potentially harmful bacteria. Previous studies have also shown that UA has corrective effect on bacterial dysbiosis by increasing the potential beneficial bacteria, such as the phylum *Firmicutes* and the genera *Lactobacillus* and *Bifidobacterium* in mice with liver fibrosis (Wan et al., 2019). Some members of the phylum Firmicutes can inhibit the growth of opportunistic pathogens and some are known to be involved in the degradation of complex carbohydrates (Brown et al., 2012). Lactobacillus, as a member of the phylum Firmicutes, plays an important role in the growth and development of the animal intestine by secreting metabolites, competitive rejection, immune regulation and other ways (Ashraf and Shah, 2014). In this study, the microflora structure of mice in the UA group might be better than that of the Tet group, which might be due to the increase of *Lactobacillus*. Shin et al. (2015) reported that an imbalanced gut microbiota arised from a sustained increase in abundance of the phylum Proteobacteria (Shin et al., 2015). In this study we showed that there was greater abundance of Proteobacteria detected in the mice of NatR group. In contrast, the UaT group, supplementation with UA from the day 15, reversed this trend.

There is no doubt that long-term use of antibiotics can lead to resistance in intestinal microflora (Bäumler and Sperandio, 2016). Previous reports had shown that adding antibiotics to the diet could make gut bacteria resistant, and the level of antibiotic use can influence the prevalence of certain drug-resistant genes (Blake et al., 2003; Price et al., 2019). The results in the present study showed that antibiotic exposure increased the number and types of TRGs in gut microbiota of mice. Quite a few literatures analyzed and discussed the influence of PEs on drug-resistant bacteria (Ginovyan and Trchounian, 2019; Sahakyan et al., 2019). Carnosic acid and carnosol were found to potentiate the activity of tetracycline (2- and 4-fold, respectively) against a TetK-possessing *S. aureus* strain (Abreu et al., 2012), and UA was found to have a good antibacterial effect against the methicillin-resistant Staphylococcus aureus (Wang et al., 2016). 46 different TRGs have been detected, and among these resistance determinants, four different bacterial mechanisms of resistance to tetracycline drugs have been identified, including efflux pump, ribosomal protection, enzyme inactivation and unknown mechanisms (Roberts and Schwarz, 2016). In our study, the TRGs expression of TET(34), TETG-01, and TETL-01 was significantly down-regulated in the UA group. Among them, TET(34) was involved in enzyme inactivation, and TETG-01 and TETL-01 were involved in the efflux pump (Roberts and Schwarz, 2016). Additionally, Wang et al. (2016) found that UA could affect the integrity of bacterial membranes (Wang et al., 2016). Thus, we deduced that efflux pump and enzyme inactivation might be the regulatory targets of UA to TRGs. Moreover, the significantly downregulated TRGs expressions [TETM-02, TETQ, TETG-01, TETR-03, TETB-02, TET(36)-01 and TETG-02] indicated that UA could enhance the binding ability of bacterial ribosomes and tetracycline to reduce drug resistance (Taylor and Chau, 1996; Li et al., 2013). Taken together, UA could down-regulate the expression of TRGs through various mechanisms.

## Conclusion

At present, there are few studies on ursolic acid in mice, and the selection of the number of mouse samples in our experiment also has some limitations. More studies are needed to confirm the effects of ursolic acid on mice. In summary, the results of the present study demonstrated that UA had positive effects on the growth performance of mice. More importantly, it significantly improved the intestinal mucosal barrier in mice with antibiotic exposure by promoting intestinal nutrient absorption and tight junction proteins expression, and inhibiting intestinal inflammation. Moreover, this study revealed that the influence of UA on ARGs in intestinal microflora and demonstrated that UA effectively lowered the expression of antibiotic-induced resistance genes. Considering the positive effects of UA on the growth performance and intestinal mucosal barrier, we anticipate that these findings could be a stepping stone for developing UA as a novel substitute of antibiotics in animal industries.

## Data Availability Statement

The datasets presented in this study can be found in online repositories. The names of the repository/repositories and accession number(s) can be found below: NCBI SRA BioProject, accession no: PRJNA705824.

## Ethics Statement

The animal study was reviewed and approved by the Hunan Agricultural University.

## Author Contributions

FP designed the experiment, conducted the research, and wrote the manuscript. HZ and ZS revised the article. XH analyzed the data. All authors contributed to the article and approved the submitted version.

## Conflict of Interest

The authors declare that the research was conducted in the absence of any commercial or financial relationships that could be construed as a potential conflict of interest.

## Footnotes

1 http://www.wcgene.com
