# Supplementary material for: Effects of Ursolic Acid on Intestinal Health and Gut Bacteria Antibiotic Resistance in Mice
Source: Front Physiol. 2021 May 28;12:650190. doi: 10.3389/fphys.2021.650190 (PMC8195277; doi:10.3389/fphys.2021.650190)
Supplement: Supplementary file 1 [file Table_1.DOCX]

Table S1. The composition of basal diet in mice

| **Main Raw Materials** | |
| --- | --- |
| Protein | Soybean meal, Fish meal, Beer yeast powder |
| Fat | Vegetable oil |
| Carbohydrate | Corn, Wheat |
| Fiber | Alfalfa meal, Wheat bran |
| Vitamin | VA, VD, VE, VB1, VB6, VB5, etc. |
| Mineral | Dicalcium phosphate, Rock flour, Fe, Cu, Mn, Zn, etc. |
| **Guaranteed Value of Nutrients** | |
| Water (%) ≤10 | |
| Crude protein (%) ≥18 | |
| Crude fat (%) ≥4 | |
| Crude fiber (%) ≤5 | |
| Crude ash (%) ≤8 | |
| Ca (%) 1.0-1.8 | |
| P (%) 0.6-1.2 | |
| Lysine (%) ≥0.82 | |
| Methionine + Cystine (%) ≥0.53 | |
